# Sexually-trimorphic interactions with colour polymorphism determine nectar quality in a herbaceous perennial

**DOI:** 10.1038/srep45838

**Published:** 2017-04-04

**Authors:** Sandra Varga, Carl D. Soulsbury

**Affiliations:** 1 School of Life Sciences, University of Lincoln, Joseph Banks Laboratories, Lincoln LN6 7TS, UK

## Abstract

Amongst gynodioecious plant breeding systems, there can exist intermediate morphs with a reduction in their male function (i.e. reduced number of functional anthers). Along with this sexual trimorphism, plants can also show floral colour polymorphism. Such intricate mixtures of phenotypes within a species may have complex effects on floral rewards. Floral rewards are known to vary between sexually dimorphic species and to a lesser extent between colour morphs. However, the interactive effect of sexual trimorphism and colour polymorphism is unexplored. We measured nectar’s sugar content in the sexually trimorphic *Geranium sylvaticum*, a gynodioecious plant with a light/dark floral polymorphism. We found that nectar reward differed across genders and colour morphs. Results were not however consistent within the three genders; dark female and hermaphrodite flowers had higher sugar content than light morphs, whereas intermediate flowers did not. As expected, females and hermaphrodites had different nectar reward, with intermediate morphs being midway between the other genders. In intermediates, the sugar content was not related to the number of functional stamens. We show for the first time the existence of sex-specific differences between flower gender and colour morphs in nectar rewards. Our results demonstrate the importance of considering multiple and conflicting selection pressures to explain rewards.

Together with pollen, nectar is the primary reward provided by flowers for pollinators across angiosperms^1^. Nectar composition varies widely, both quantitatively and qualitatively in response to strong selective pressures by pollinating animals^2^. At the same time, there are several secondary extrinsic and intrinsic factors that drive nectar quality. Some well-known ones include abiotic factors such as humidity^3^ and temperature^4^, as well as floral specific ones such as sexual phase of flowers^5^ or flower age^4^. However, the innumerable sources of variation may mask potentially key patterns of variation within populations.

From the perspective of pollination, the relationship between nectar quality and colour would be expected to be particularly important^1^. Most pollinators have good colour vision and show certain innate colour preferences^6^,^7^. Many also have the ability to associate different colours with different levels of nectar or pollen reward^8^,^9^. In species where more than one colour morph exists (colour polymorphism), directional selection by pollinators alone or in combination with random genetic drift should lead to the loss of floral colour polymorphism especially if different colours differed in their rewards. So far, comparatively few studies have measured colour-specific differences in floral rewards. Results have been mixed, some have found no differences in nectar quality^10^, high variability between multiple colours^11^ or differences between distinct colour morphs^12^.

From the plant perspective, the rewards offered to pollinators must balance the rewards costs *versus* the investment in reproduction^1^. Evidence of this is clearest when examining nectar rewards in relation to plant breeding systems. Most flowering plants are hermaphroditic (i.e. containing both the female and male sexual function within the same flower) but plant breeding systems with separate female and male functions in different individuals have evolved several times^13^. Numerous studies have shown that female flowers have lower rewards than hermaphrodites in gynodioecious species^14^. Similarly, in species with dichogamy, nectar quantity is typically highest during the males phase^12^. To further complicate matters, many gynodiecious species contain a third distinct phenotype^15^. This phenotype is made up of individuals with an intermediate, or partially male-sterile, phenotype (i.e. individuals with a mixture of pistillate and perfect flowers or with mixed flower types). These individuals are not uncommon and typically have a frequency in natural populations comparable or higher to that of females^15^. One major issue is that in studies of gynodioecy, treatment of partially male-sterile individuals is variable. Sometimes they have been excluded from the analysis^16^, included in the male-sterile category^17^, or included in the hermaphrodite category because they produce some pollen^18^. This may be problematic, as it ignores the possible cost of a male-fertility restoration and particularly how this impacts floral traits.

Combined, there are number of key pressures that act on nectar rewards. Most have been examined in isolation (e.g. comparing colour morphs^19^; or genders, reviewed in^14^), which does not adequately represent the complex factors impacting nectar quality. In the present study, we use the gynodioecious plant, *Geranium sylvaticum* (wood cranesbill), as a model species to examine the combined effect of abiotic and floral-specific traits on nectar’s sugar content and production. *G. sylvaticum* is a widely distributed Eurasian perennial plant with visible and UV-spectral floral colour polymorphism. Petal colour ranges from white to deep purple^20^, even though in Finland most populations are composed by pink and purple morphs. *G. sylvaticum* is sexually trimorphic; most populations are gynodioecious^21^ and contain in addition to female and hermaphrodite (i.e. individuals with ten functional stamens per flower) plants, intermediate individuals^22^. Three types of flowers are recognised depending on the number of functional stamens: pistillate (referred as female flowers hereafter) and perfect (referred as hermaphrodite flowers hereafter) possess 0 and 10 stamens respectively, and intermediate flowers possess one to nine functional anthers and one to nine staminoides.

Investigating whether nectar quality varies differentially between floral genders for the two colour morphs is fundamental to predict whether pollinators may exert a selective pressure on this factor. Because colour polymorphism is observed in most *G. sylvaticum* populations, we hypothesised that the two colour morphs would not differ substantially in nectar rewards. Moreover, we expected the hermaphrodite flowers to produce higher nectar rewards in line with previous reports, regardless of the floral colour.

## Materials and Methods

### Experimental design

**Figure 1:**
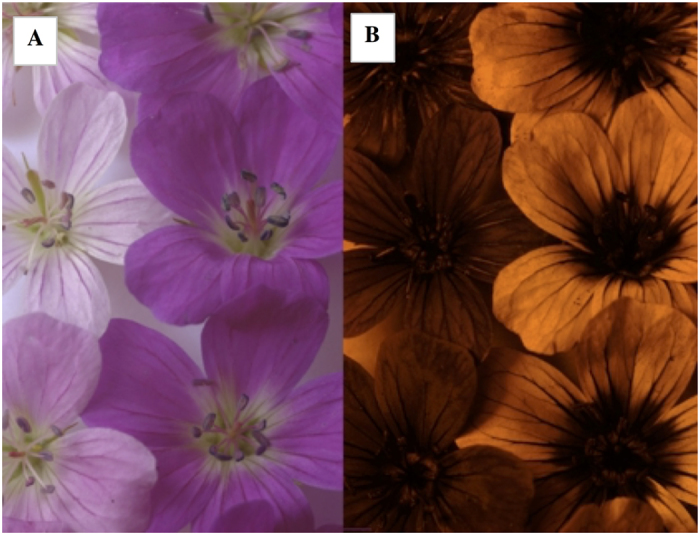
Light and dark colour morphs of *Geranium sylvaticum* under (**A**) natural and (**B**) from UV photography.

Full experimental details can be found in^5^. Briefly, during summer 2008, we measured sugar quantity (referred as sugar content hereafter) in nectar samples randomly collected from 103 female, 157 intermediate and 313 hermaphrodite flowers (*N* = 573) within a wild population growing at Oulu University Botanical Gardens, Finland (65°03’N, 25°27’E). Samples were collected at the beginning of the peak of maximum pollinator activity (1100 h, *N* = 277) or after (1500 h, *N* = 296) from flowers protected from pollinators using small mesh bags (*N* = 234) or left untouched (*N* = 339) and thus available to pollinators. Nectar was extracted from freshly cut flowers with paper wicks as described in^23^. Sugar content was determined using the anthrone method^24^, and absorbance read at 620 nm with a BioSpec-1610E spectrophotometer (Shimadzu, Kyoto, Japan). We measured total sugar content as it was not possible to estimate nectar volume from the flowers. Moreover, this parameter has been shown to influence pollinator visitation and it is used to examine the costs and benefits of resource allocation to pollinator attraction in sexually dimorphic plants (i.e.^25^,^26^. For each flower, we categorised the colour as dark (purple flowers) or light (white/pink flowers), based on a clear difference in the UV spectra (Fig. 1). We noted the floral stage for each flower (see^5^). Air temperature and humidity at the time of the samplings were obtained from the Finnish Meteorological Institute (http://en.ilmatieteenlaitos.fi).

### Statistical analyses

We set out to examine the effect of flower colour (light/dark) and gender (female/intermediate/hermaphrodite) on nectar’s sugar content. Sugar content (in *μg*) was 10 + log-transformed before analysis to correct for left-skewness in residuals and to meet the model assumptions of normality and heteroscedasticity. We included in the model other potentially relevant variables know to impact nectar’s sugar content: (a) number of nectaries with visible nectar at the time of sampling, (b) time of nectar collection (morning/afternoon), (c) floral stage was fitted as a factor (1 = non-receptive for pollination, i.e. when no pollen is exposed, and/or the five stigmatic lobes remained closely joined to each other, 2 = male phase, i.e. with stamens exposing pollen, 3 = female phase, i.e. the five stigmatic lobes are unfolded and exposing the papillate stigmatic surfaces becoming receptive for pollen), (d) relative humidity (%), (e) log temperature at sampling (°C), (f) treatment (bagged/unbagged), (g) sampling day, (h) total rainfall (mm) in previous 24 hours. Relative humidity, temperature and rainfall were included as covariates. In the full model, we included the interactions between colour × sex and time × treatment.

We initially ran a full model with all single predictors and no interactions and tested for collinearity among fixed factors using variance inflation factor (VIF) values from the car package^27^. Two factors (day and temperature) had VIF values above the recommended upper threshold value of 3^28^. As a result, we excluded day of nectar collection as a predictor variable from the full model. We then ran a full general linear model. Where interactions or categorical variables were significant, we carried out *post hoc* pairwise comparisons comparing within-sex (colour × sex) and between times (time × treatment) using unadjusted least-square means. *Post hoc P* values were adjusted for multiple testing with Tukey’s correction.

Based on previous studies’ lack of examination of intermediate phenotypes, we examined how nectar’s sugar content differed in relation to investment in male function (number of functional stamens). We built the same full model as before, but without gender as a factor and including the number of functional stamens as a predictor variable. Subsequently, we carried out *post hoc* pairwise analysis where interactions or categorical variables were significant using unadjusted least-square means.

All statistical analyses were run in R version 3.2.1 using *lm* function, with *post hoc* analysis carried out using the lsmeans package^29^.

## Results

### Gender-colour morph interaction on nectar quality

**Figure 2:**
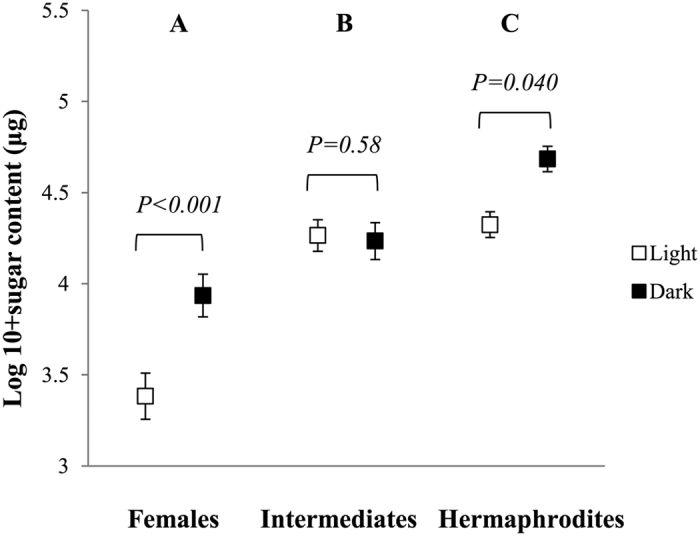
Mean ± 1SE sugar content (in *μg*, log-transformed) in nectar sampled from female, intermediate and hermaphrodite light (white boxes) and dark (black boxes) *Geranium sylvaticum* flowers. *Post hoc* statistical significant differences (*P* < 0.05) between genders are indicated with different letters.

Sugar content in the nectar samples ranged between 0 and 1120 μg (average ± SE, 105.6 ± 5.7 μg). There was a significant interaction between gender and colour (F_2,558_ = 4.27, *P* = 0.014). In female (β ± SE = −0.50 ± 0.14, t-ratio = −3.43, *P* < 0.001) and hermaphrodite (β ± SE = −0.17 ± 0.08, t-ratio = −2.01, *P* = 0.040) flowers, dark morphs had higher sugar content in their nectar, whereas in intermediate flowers there was no difference between the two colour morphs (β ± SE = −0.06 ± 0.12, t-ratio = 0.55, *P* = 0.58; Fig. 2). Irrespective of colour, there were significant differences between genders (F_2,558_ = 32.66, *P* < 0.001). Females had significant lower sugar content than both intermediates (β ± SE = −0.49 ± 0.09, t-ratio = −2.52, *P* = 0.027) and hermaphrodites (β ± SE = −0.26 ± 0.10, t-ratio = −5.21, *P* < 0.001), and intermediates had lower sugar content than hermaphrodites (β ± SE = 0.23 ± 0.07, t-ratio = 3.23, *P* = 0.004; Fig. 2).

### Floral traits and nectar quality

**Figure 3:**
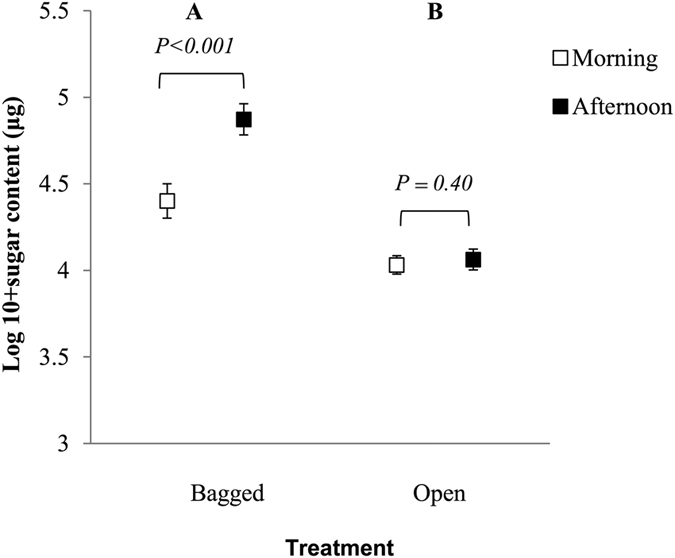
Mean ± 1SE sugar content (in μg, log-transformed) in nectar sampled from bagged (white boxes) and unbagged (black boxes) *Geranium sylvaticum* flowers. *Post hoc* statistical significant differences (*P* < 0.05) between treatments are indicated with different letters.

There was a significant interaction between time and treatment (F_1,558_ = 15.19, *P* < 0.001; Fig. 3). For bagged flowers, there was a higher sugar content in nectar collected in the afternoon (β ± SE = −0.41 ± 0.09, t-ratio = −4.20, *P* < 0.001), but not for open flowers (β ± SE = 0.07 ± 0.08, t-ratio = 0.84, *P* = 0.40; Fig. 3). In general, nectar’s sugar content was higher in the afternoon (F_1,558_ = 8.71, *P* = 0.003) and in bagged flowers (F_1,558_ = 69.74, *P* < 0.001).

**Figure 4:**
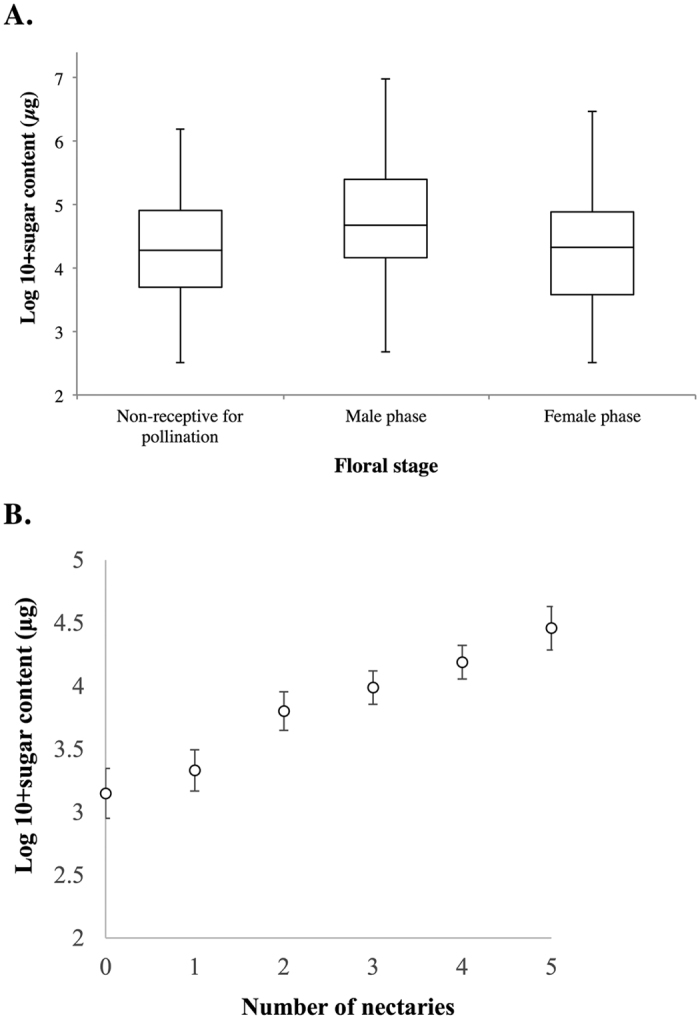
(**A**) Boxplot showing the relationship between nectar sugar content (in *μg*, log-transformed) and floral stage and (**B**) nectar sugar content (in *μg*, log-transformed) and number of nectaries with visible nectar.

There was also a significant effect of floral stage on nectar’s sugar content (F_2,560_ = 11.41, *P* < 0.001); this is not surprising as the male (β ± SE = −0.37 ± 0.07, t-ratio = −4.75, *P* < 0.001) and female (β ± SE = −0.24 ± 0.08; t-ratio = −2.92, *P* = 0.010) stage is known to have greater sugar content compared to stage 1 (Fig. 4A). However, the male and female stages did not differ significantly from each other (β ± SE = 0.13 ± 0.08, t-ratio = 1.61, *P* = 0.24). In addition, the number of nectaries was positively correlated with sugar content (β ± SE = 0.41 ± 0.10, F_1,558_ = 23.75, *P* < 0.001; Fig. 4b).

### Abiotic factors and nectar quality

**Figure 5:**
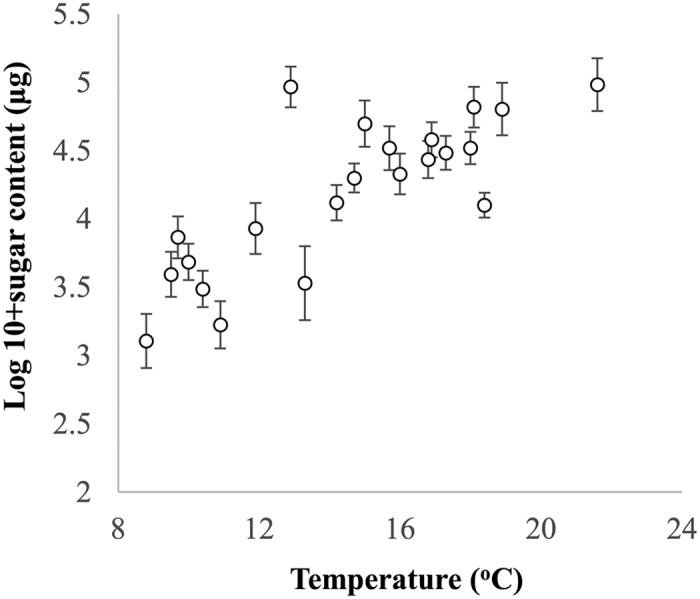
Relationship between nectar sugar content (in *μg*, log-transformed) and temperature. Values represent mean ± SE.

There was a significant positive effect of temperature on amount of sugar in nectar (β ± SE = 1.52 ± 0.017, F_1,558_ = 193.07, *P* < 0.001; Fig. 5). Interestingly there was a positive effect of relative humidity on nectar’s sugar content in the full model (β ± SE = 0.01 ± 0.00, F_1,558_ = 5.67, *P* = 0.017). Rainfall in the previous 24 hours had no effect on the sugar content of nectar (β ± SE = 0.05 ± 0.03, F_1,558_ = 1.24, *P* = 0.27).

Overall the full model was highly significant (F_14, 558_ = 30.03, *P* < 0.001) and explained a moderate amount of variance in the data (R^2^ = 0.415).

### Intermediate flowers and investment in nectar sugar content

Similar floral and abiotic factors effected sugar content in nectar within intermediate flowers. It positively related to the number of nectaries (β ± SE = 0.16 ± 0.05, F_1,139_ = 10.78, *P* = 0.001), temperature (β ± SE = 2.32 ± 0.39, F_1,139_ = 57.04, *P* < 0.001) and was significantly lowest in unbagged flowers (β ± SE = −0.47 ± 0.16, F_1,139_ = 32.56, *P* < 0.001). There was also a significant effect of floral stage (F_2,139_ = 6.75, *P* = 0.002); the male stage (β ± SE = −0.37 ± 0.14, t-ratio = −2.66, *P* = 0.024), but not the female stage (β ± SE = −0.16 ± 0.15; t-ratio = −1.12, *P* = 0.51) had greater sugar content than stage 1. The male and female stages did not differ significantly (β ± SE = 0.20 ± 0.14, t-ratio = 1.46, *P* = 0.31).

In contrast, sugar content was unrelated to colour in intermediate flowers (β ± SE = −0.05 ± 0.11, F_1,139_ = 0.09, *P* = 0.77), relative humidity (β ± SE = 0.01 ± 0.01, F_1,139_ = 0.03, *P* = 0.86) and rainfall (β ± SE = 0.00 ± 0.06, F_1,139_ = 0.02, *P* = 0.90). There was a trend for higher sugar content in the afternoon (β ± SE = 0.27 ± 0.19, F_1,139_ = 3.46, *P* = 0.07) and the interaction with treatment (F_1,139_ = 1.73, *P* = 0.19). However, there was no relationship between sugar content and number of functional stamens (β ± SE = 0.00 ± 0.02, F_1,139_ = 0.02, *P* = 0.88).

Overall the full model was highly significant (F_11,139_ = 10.84, *P* < 0.001) and explained a moderate amount of variance in the data (R^2^ = 0.421).

## Discussion

### Gender differences on nectar quality

Our results confirm the previously reported patterns of sexual dimorphism in sugar production in gynodioecious plants, where female flowers have lower nectar quality than hermaphrodites^5^,^14^. Moreover, we show for the first time that intermediate flowers have different patterns of nectar sugar content from the other two genders, but patterns of production that are similar to hermaphrodites and not to females. Greater sugar content and production in hermaphrodite flowers is predicted from sexual selection theory^30^, which assumes that male fitness is most strongly limited by access to mates. Therefore, hermaphrodite flowers should invest relatively more in the production of nectar reward than females. Similarly, intermediate flowers produce some pollen and would be expected to invest more into rewards than female flowers. At the same time, the investment in male function and the duration of the male phase in intermediate flowers is shorter than in hermaphrodite flowers in some species^31^, so it could be predicted that investment in nectar rewards should be lower. Clearly, a more detailed examination of why nectar rewards in intermediates differ from female and hermaphrodite flowers is critically needed, to more fully understand the mechanisms driving this variation.

### Colour differences on nectar quality

In contrast to previous studies, our results also demonstrate that overall, darker (purple) flowers had higher amounts of sugar than lighter (pink) morphs. Most studies report no difference between colour morphs^10^,^11^, but one study found that lighter morphs (white) had better nectar quality than dark (pink) morphs^12^. Petal colouration can affect flower temperature^32^. It is generally believed that darker flowers are warmer, though evidence from wild studies is mixed^32^,^33^ probably because there are several selective forces acting on petal colouration and flower thermogenesis (such as phylogenetic constraints, or due to the role of flower pigmentation in stress tolerance, e.g.^34^). In turn, warmer flowers tend to produce more nectar of higher sugar concentration than that of cooler flowers^35^. Differences, or lack of between colour polymorphism, may therefore stem from the nature of the floral polymorphism. For example, clear differences between colours (e.g. this study;^12^) may contrast with those with a gradient of colour morphs (e.g. light blue to purple^10^; white-pink-purple^11^). Clearly, a better understanding of the type of differences, their selection and maintenance is needed when comparing colour-specific differences within species.

### Floral and abiotic factors affecting nectar quality

Our work demonstrates the complexity of studying nectar quality. It is well known that many variables act on determining the amount and quality of nectar available in a flower (see^36^, and references there). Similarly, our work demonstrates that abiotic (temperature, relative humidity), temporal (time of day) and floral-specific patterns (gender, floral stage, number of visible nectaries with nectar) are all impacting nectar quality simultaneously. Indeed, the only factor that did no impact sugar content of nectar was rainfall. Other studies that experimentally watered plants have shown an increase in sugar concentration^37^, especially under conditions of water stress^38^. In our population, it is very unlikely that plants were water stressed so other abiotic factors (relative humidity, temperature) were more important.

### Intermediate phenotype and nectar quality

Our results of colour-specific differences were not consistent within the third sexual phenotype i.e. intermediate flowers. In this case, the amount of sugar was similar in both colour morphs, and contrasted with the patterns observed in female and hermaphrodite flowers. Partial male sterility is widespread in gynodioecious species, and the loss of the male function may occur in varying degrees, from complete abortion of the entire anther to nuclear abnormalities at pollen grain mitosis^39^. Intermediates in most gynodioecious species typically show vegetative and reproductive characters that are midway between females and hermaphrodites, including our study species (reviewed by^22^). Our results support this, with sugar content being midway between both genders, being higher than females and lower than hermaphrodites.

Anther development can be divided into two general phases: phase one in which the anther morphology is established, cell and tissue differentiation occur, and microspore mother cells undergo meiosis; and phase two, in which pollen grains differentiate^40^. The presence of aborted stamens is evidence of failure during the early ontogeny of stamens (phase one). At the same time, the proportion of fertile pollen grains is typically lower in intermediates (20,41), indicating that there is additional failure at phase two. Such a pattern suggests some possible positive or antagonistic pleiotropic effects through genetically correlated characters^42^, which perhaps link together male restorer genes and floral traits^43^,^44^. Alternatively, the costs of male sterility restoration^45^,^46^, may be particularly important in intermediates. In either case, the relationship between sugar content in a number of variables including colour appears to be disrupted or weaker.

### Data accessibility

The dataset supporting this article will be uploaded to Data Dryad upon acceptance.

## Additional Information

**How to cite this article:** Varga, S. and Soulsbury, C. D. Sexually-trimorphic interactions with colour polymorphism determine nectar quality in a herbaceous perennial. *Sci. Rep.* **7**, 45838; doi: 10.1038/srep45838 (2017).

**Publisher's note:** Springer Nature remains neutral with regard to jurisdictional claims in published maps and institutional affiliations.
